# Spatial–temporal variations in deforestation hotspots in Sumatra and Kalimantan from 2001–2018

**DOI:** 10.1002/ece3.7562

**Published:** 2021-05-02

**Authors:** Minerva Singh, Siheng Yan

**Affiliations:** ^1^ Centre for Environmental Policy Imperial College London London UK

## Abstract

Tropical deforestation varies temporally and spatially which can inhibit the ability of existing protected areas to stem forest loss. Identifying the spatial–temporal distribution of deforestation and its concentration can help decision makers decide conservation priorities and leverage limited resources. This study assessed how topographic and anthropogenic variables affect deforestation patterns within and outside protected areas on the islands of Sumatra and Kalimantan in Indonesia. Emerging hotspot analysis (EHA) was used to evaluate spatial and temporal trends of forest loss on the Hansen annual forest loss data for these islands from 2001–2018. For the two islands, most hotspots were detected outside protected areas; those within protected areas were mainly concentrated at boundaries, where lower elevation/slope and high human pressure could be observed. New hotspots were identified within the three PAs in Sumatra, while three kinds of hotspots (consecutive, oscillating, and sporadic) were found in the two PAs of Kalimantan (Kutai and Teluk Kelumpang). Areas with high human pressure (average human footprint higher than 12) were covered by a high density of hotspots. The results identify specific areas where forest loss has emerged recently, which could indicate a conservation priority. It is suggested that new protected areas be established in locations showing intensifying and persistent hotspots—those where deforestation has occurred for ≥16 of 18 years of the study period.

## Graphical abstract

Emerging hotspot analysis, a new approach that can evaluate spatial and temporal trends of forest loss, was performed on annual forest loss data for these islands from 2001–2018. Most hotspots were detected outside PAs; those within PAs were mainly concentrated at boundaries, where lower elevation/slope and high human pressure could be observed. More hotspots were detected on Sumatra than Kalimantan and were mainly distributed in the island's center

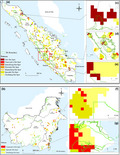

## 1 INTRODUCTION

Indonesia—the third largest extent of tropical forests in the world—experienced high deforestation of over 6 million hectares from 2000 to 2012, occurring mainly in the Sumatra and Kalimantan islands (Margono et al., 2014). Sumatra lost 68% of its forest in eastern provinces between 1990 and 2010 (Margono et al., 2012); Kalimantan's lowland protected forests declined by 56% from 1985 to 2001 (Curran et al., 2004). Large‐scale oil palm establishments, followed by timber plantations, are a leading cause of deforestation in Indonesia (Austin et al., 2019). From 1995–2015, oil palm expansion occurred at an average rate of 450,000 ha/yr and resulted in an average of 117,000 ha/yr of deforestation (Austin et al., 2017). Since 1989, 45% of the region's oil palm plantations have been developed on previously forested land compared to 2% in South America (Vijay et al., 2016).

Over the past few decades, Sumatra has especially seen high rates of deforestation, driven by an expansion in oil palm plantations (Austin et al., 2019; Gaveau et al., 2016; Koh & Wilcove, 2008). From 1973–2015, Kalimantan lost an estimated 14.4 million hectares of old‐growth forests (Gaveau et al., 2016). Conversion to oil palm plantations remains a leading cause of deforestation in Kalimantan as well (Sumarga & Hein, 2016). However, the patterns of oil palm expansion and its impacts vary across the different provinces of Indonesia, including between Sumatra and Kalimantan (Austin et al., 2017).

**FIGURE 1:**
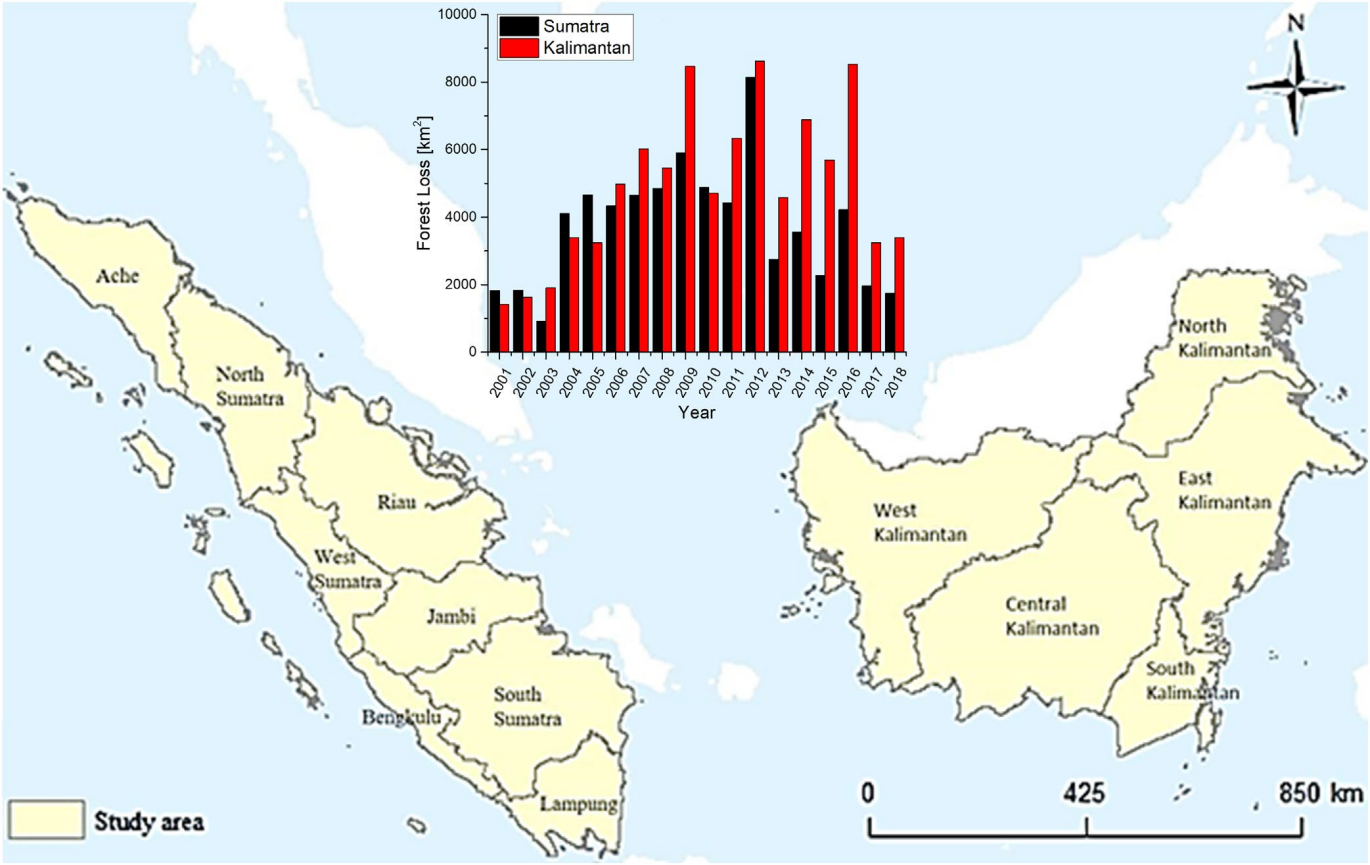
Location of the study area: Sumatra (left) and Kalimantan (right) in Indonesia and their corresponding deforestation rates from 2001 to 2018 (inset) (Hansen et al., 2013)

Figure 1 shows the location of Sumatra and Kalimantan in Indonesia, with the inset showing the annual rate of deforestations on these islands from 2001–2018 based on the Hansen Global Forest Change (Hansen et al., 2013). Over the span of 18 years, Sumatra has lost a total of 67,104 km^2^, averaging 3,728 km^2^ per year. Kalimantan has lost more during this span, with a total of 88,504 km^2^ of deforestation and averaging 4,916 km^2^.

Describing the temporal and spatial distribution of the deforestations is crucial in mitigating the deforestation in the islands.

Protected areas (PAs) have been widely recognized as a bulwark against deforestation in Indonesia (Brun et al., 2015). The IUCN categories for protected areas and descriptions are summarized in Table 1.

**TABLE 1:** IUCN categories of protected areas (Dudley, 2008)

Categories	Description
Ia	Strict Nature Reserve: Category Ia areas are strictly protected areas with biodiversity and possible geological/geomorphic features and where human visitations, uses, and impacts are strictly controlled. Such protected areas are indispensable reference areas for scientific research and monitoring.
Ib	Wilderness Area: Category Ib areas are protected for long‐term ecological integrity of natural areas that are undisturbed by significant human activity, free of modern infrastructure and where natural forces and processes predominate.
II	National Park: Category II protected areas are large natural or near natural areas, large‐scale ecological processes (with species and ecosystems characteristic of the area), that provide a foundation for environmentally and culturally compatible, spiritual, scientific, educational, recreational, and visitor opportunities.
III	Natural Monument or Feature: Category III areas are protected for a specific natural monument landform, sea mount, submarine cavern, and geological feature (cave or ancient grove). These are generally quite small protected areas and often have high visitor value.
IV	Habitat/Species Management Area: Category IV areas are protected for particular species or habitats. Many Category IV protected areas need regular, active interventions to address the requirements of particular species or habitats.
V	Protected Landscape/Seascape: Category V protected areas have interaction of people and nature over time that produced an area of distinct character with significant, ecological, biological, cultural, and scenic value: and where safeguarding the integrity of this interaction is vital to protecting and sustaining the area and its associated nature conservation and other values.
VI	Protected area with sustainable use of natural resources: Category VI protected areas conserve ecosystems and habitats including associated cultural values and traditional natural resources. These are generally large areas: Most are in a natural condition; a proportion is under sustainable natural resource management; and low‐level nonindustrial use of natural resources compatible with nature conservation is seen as one of the main aims of the area.
Not reported	For PAs where an IUCN category is unknown and/or the data, the provider has not provided any related information.
Not applicable	The IUCN Management Categories are not applicable to a specific designation type.

PAs, though, are still vulnerable to human encroachment and subsequent deforestation (Dudley, 2008). Socioeconomic aspects (demand for commodities) (Prabowo et al., 2017) and physical environment (accessibility) (Poor, Frimpong, et al., 2019, Poor, Jati, et al., 2019) drive deforestation. For instance, the demand for palm oil has caused significant forest cover change (more than 29,000 km^2^) in lowland PAs of Kalimantan from 1985 to 2001 (Curran et al., 2004). Kalimantan's PAs have faced increasing forest loss as a result of elevation and anthropogenic disturbances (Harris et al., 2008) along with a shift in oil palm expansion from Sumatra to Kalimantan (Austin et al., 2017). However, upland and hard‐to‐reach forests have lower risks to deforestation due to lower human influence stemming from lower population densities and topographic difficulties (Nugroho et al., 2018).

Patterns of deforestation vary both in terms of spatial–temporal distribution of forest loss, along with changes in the intensity of this phenomenon. So, deforestation can vary between different regions and change in intensity and location with time (Portillo‐Quintero & Smith, 2018; Reddy et al., 2019). Mapping and quantifying these spatial–temporal changes are important for informing conservation management.

Emerging hotspot analysis (EHA) incorporates temporal trends in the spatial distribution for examining the spatial–temporal in patterns relating to deforestation (Reddy et al., 2019), fire activity (Reddy et al., 2019, 2020), disease (Karunaweera et al., 2020), and rainfall patterns (Marumbwa et al., 2019). EHA is underpinned by a space–time pattern mining paradigm within a geographic context that help examine the complex data trends that occur across a landscape over time (Portillo‐Quintero & Smith, 2018; Reddy et al., 2020). EHA‐based spatial–temporal hotspot analysis was previously used to identify the changes of forest loss patterns across the tropical dry forest ecosystems of Central America as result of anthropogenic pressures. The study identified the presence of stable low‐density tropical dry forest (TDF) forest loss in Mexico and the prevalence of increasing forest loss at different spots in Central America, including the southern Yucatan peninsula (Portillo‐Quintero & Smith, 2018). EHA effectively categorizes the spot distribution using eight specific trends: new, consecutive, intensifying, persistent, diminishing, sporadic, oscillating, and historical. This tool was useful in detecting deforestation trends in tropical countries, namely India from 1982 to 2015 (Duraisamy et al., 2018); Democratic Republic of Congo from 2000 to 2014 (Harris et al., 2017); Amazonia from 2001 to 2014 (Kalamandeen et al., 2018); and Colombia from 2002 to 2010 (Sanchez‐Cuervo & Aide, 2013). Recent studies had shown the effective use of machine learning in spatio‐temporal hotspot analysis. It was used to support the search for factors with a spatio‐temporal correlation to dengue outbreaks (Anno et al., 2019), soil erosion (Chakrabortty et al., 2020), and crime prediction (Hajela, 2020).

In this study, the topographic and anthropogenic variables were assessed on how they affect deforestation patterns within and outside protected areas on the islands of Sumatra and Kalimantan in Indonesia. Specifically, EHA was used to identify the spatial–temporal variations in deforestation hotspots in Sumatra and Kalimantan from 2000–18. Lastly, the role of common deforestation drivers, such as those related to topography and anthropogenic disturbances, in explaining the different spatial–temporal patterns of deforestation in Sumatra and Kalimantan was established using machine learning.

## 2 MATERIALS AND METHODS

Deforestation hotspots in Sumatra and Kalimantan from 2001–2018 based on the Hansen Global Forest Change were investigated using EHA. Explanatory variables such as elevation and slope, oil palm and wood fiber plantation, and human footprint were assessed as to their importance in the formation of these hotspots using decision trees. Hotspot maps for Sumatra and Kalimantan were evaluated by overlaying with confounding variables to see how they might affect the spatial patterns of hotspots.

### 2.1 Study area

Sumatra—the second‐largest island (473,481 km^2^) in western Indonesia—is bordered by the Indian Ocean to the west and Straits of Malacca to the northeast and divided into eight administrative provinces: (from north to south) Aceh, North Sumatra, Riau, West Sumatra, Jambi, Bengkulu, South Sumatra, and Lampung (Figure 1, left). Kalimantan—Indonesian portion (73%–544,150 km^2^) of the island of Borneo—is bordered by the Sulawesi Sea to the northeast, Makassar Strait to the east, and Java sea to the south (Figure 1, right).

### 2.2 Data

#### 2.2.1 Forest data

Landsat images (30‐m spatial resolution), from 2001 to 2018, were processed to extract the Global Forest Change (GFC) deforestation data (Hansen et al., 2013). The data were encoded at values 0–18 for the time period considered in the study (Harris et al., 2017).

#### 2.2.2 Protected areas

Terrestrial protected areas in Sumatra and Kalimantan were taken from Protected Planet database (UNEP‐WCMC and IUCN, 2019).

#### 2.2.3 Explanatory variables

Spatial patterns of deforestation are influenced by both topographic and anthropogenic variables (Fuller et al., 2004; Gaveau et al., 2009; Poor, Frimpong, et al., 2019; Poor, Jati, et al., 2019).

Topographic variables considered are elevation and slope, which was said to be a protection for forests from deforestation (Nüchel et al., 2019). Elevation and slope data were extracted from a digital elevation model of the earth of Shuttle Radar Topography Mission (Rabus et al., 2003).

Anthropogenic variables considered are oil palm and wood fiber plantation, and human footprint. Oil palm and wood fiber plantations have been identified as among the biggest drivers of deforestation in Sumatra and Kalimantan (Abood et al., 2015). Spatial locations of oil palm and wood fiber plantations over the study area were accessed using Global Forest Watch (World Resources Institute, 2002) and were processed using the “Near” tool, based on Euclidean distance in ArcGIS10.2 (Phompila et al., 2017). Land‐use change data were not used in this research. Kruskal–Wallis nonparametric test was also conducted to check the statistically significant differences between the variables used for explaining the variation in hotspots (Singh et al., 2019).

Global human footprint is a cumulative measure of human influence based on eight global human pressures (Venter et al., 2016a, 2016b). These data were obtained at a spatial resolution of 1km from the Socioeconomic Data and Applications Center (SEDAC) (https://sedac.ciesin.columbia.edu/). These human pressures include population density, roads, built area, pastureland, and night lights among others (Riggio et al., 2020). Many of these pressures such as roads and pasture lands are harder to detect by space‐borne satellites. Cumulative threat mapping approach adopted by Venter et al. (2016a, 2016b) aims to surmount this limitation by including a range of human pressures within a framework that couples top‐down remote sensing with data collected bottom‐up via surveys (Venter et al., 2016a, 2016b) and account for the fact these stressors often act in conjunction with each other (Williams et al., 2020).

Decision trees are a machine‐learning algorithm that use a tree‐like structure of decisions. The algorithm creates decision rules that recursively split the independent variables into homogenous zones in the form of a hierarchical model (Lee & Lee, 2015). The purpose of these recursive explanatory variables splits is to explain how the different explanatory variables explain response variable values. Decision tree creation aims to minimize the Gini coefficient (degree of inequality in a distribution) and cross‐entropy index (difference between two probability distributions for a given random variable). The initial decision tree partitions are again split into further partitions that minimize the same indices. This goes on until the degree of minimization becomes very minute, or when a prespecified stopping condition is met (Choi et al., 2018). Decision trees do not need any input preprocessing such as data normalization, scaling, or centering, and decision trees are built using predictors that have the maximum information (Alcolea et al., 2020).

### 2.3 Emerging hotspot analysis

Emerging hotspot analysis (EHA) evaluates both spatial and temporal trends of deforestation by applying two statistical methods: Getis‐Ord Gi* and Mann–Kendall. The Getis‐Ord Gi* statistic measures the trends in spatial clustering of forest loss (counts in a bin relative to its neighborhood) and provides z‐scores and p‐values (measures of statistical significance for hotspots and cold spots) (Getis & Ord, 2010; Ord & Getis, 1995). We only considered hotspots of deforestation because they have statistical significance. A hotspot with a z‐score higher 1.96 is a statistically significant (at a significance level of *p* < .05) and has a higher clustering intensity. The neighborhood distance was 10 km, and the neighborhood timestep interval (the number of time step intervals included in the analysis) was set one year since the forest data were collected annually (Harris et al., 2017).

The Mann–Kendall statistic measures the significant trend in each bin during the study period. The trend for each bin is displayed as a z‐score (positive for increasing trend; negative for decreasing trend) and a p‐value (measures whether each trend is statistically significant). The expected value of z‐score is 0 (no trend) and compared with the observed value to check statistical significance. As defined in Table 2, eight types of hotspot patterns can be generated from emerging hotspot analysis: new, consecutive, intensifying, persistent, diminishing, sporadic, oscillating, and historical (Harris et al., 2017).

**TABLE 2:** Category of eight hotspot patterns and their definitions (Harris et al., 2017)

Hotspot pattern	Definition
New	A location that is a statistically significant hotspot for the final time step and has never been a statistically significant hotspot before.
Consecutive	A location with a single uninterrupted run of statistically significant hotspot bins in the final time step intervals. The location has never been a statistically significant hotspot prior to the final hotspot run and less than ninety percent of all bins are statistically significant hotspots.
Intensifying	A location that has been a statistically significant hotspot for ninety percent of the time step intervals, including the final time step. In addition, the intensity of clustering of high counts in each time step is increasing overall and that increase is statistically significant.
Persistent	A location that has been a statistically significant hotspot for ninety percent of the time step intervals with no discernible trend indicating an increase or decrease in the intensity of clustering over time.
Diminishing	A location that has been a statistically significant hotspot for more than ninety percent of the time step intervals (for this study, 16 of the 18 years), including the final step. In addition, the intensity of clustering in each time step is decreasing overall and that decrease is statistically significant.
Sporadic	A location that is an on‐again then off‐again hotspot. Less than ninety percent of the time step intervals have been statistically significant hotspots and none of the time step intervals have been statistically significant cold spots.
Oscillating	A statistically significant hotspot for the final time step interval that has a history of also being a statistically significant cold spot during a prior time step. Less than ninety percent of the time step intervals have been statistically significant hotspots.
Historical	The most recent time period is not hot, but at least ninety percent of the time step intervals have been statistically significant hotspots.

We only displayed hotspot patterns because these are the areas that would require forest conservation. Hotspot maps for Sumatra and Kalimantan were evaluated by overlaying with confounding variables to see how they might affect the spatial patterns of hotspots.

## 3 RESULTS

### 3.1 Emerging hotspots across Sumatra and Kalimantan

**FIGURE 2:**
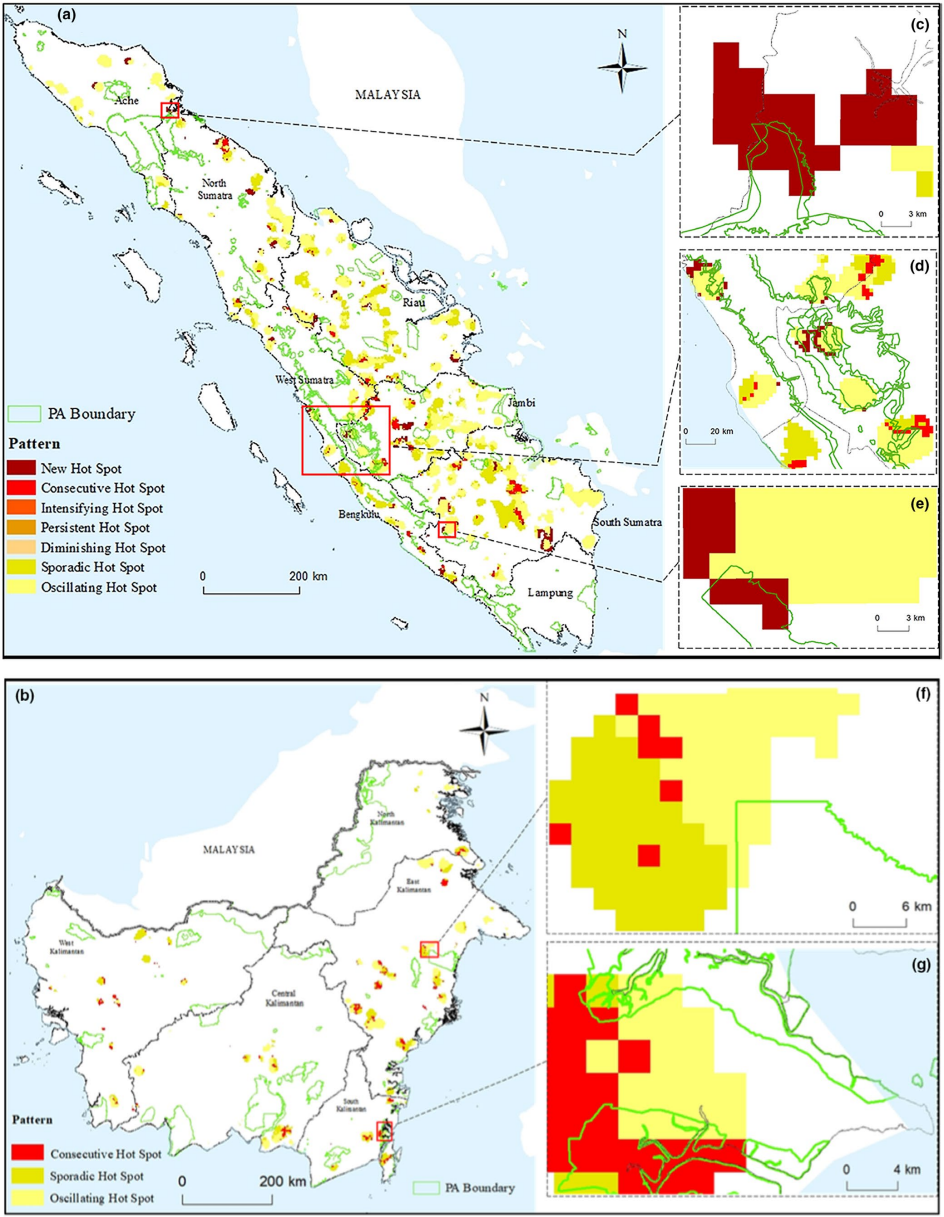
Emerging hotspot map of forest loss overlaid with locations of all preserved areas in (a) Sumatra and (b) Kalimantan. Hotspots identified in (c) Gunung Leuser National Park (IUCN category not applicable); (d) Kerinci Seblat (IUCN category II); (e) Gumai Pasemah (IUCN category IV); (f) Kutai (IUCN category II); (g) Teluk Kelumpang Selat Laut Selat Sebuku (IUCN category Ia)

Figure 2a shows the periodical deforestations (oscillating–58.8%; sporadic–29.8%), from 2001 to 2018, mostly found in the central part of Riau, Jambi, and South Sumatra. All provinces, except Lampung, have areas with high deforestation than average in 2018 (new hotspot–6.2%). These suggest that most hotspot locations in Sumatra did not consistently experience higher deforestation than their surroundings during the study period.

Only three hotspots patterns (oscillating and sporadic–85%; consecutive–15%) were detected in Kalimantan, all concentrated in the eastern and coastal regions (Figure 2b). Consecutive hotspots suggest that these locations had continuously higher forest loss than their surroundings throughout the study period. No new hotspots were observed in Kalimantan for the study period.

### 3.2 Emerging hotspots within protected areas

Majority of the hotspots on both islands developed outside PAs (Figure 2). The hotspots within the PAs were observed near the boundaries, and none covered an entire protected area (Figure 2c–g). New hotspots were identified within PAs of Sumatra (Table S1): Gunung Leuser National Park (0.26%, IUCN category II, Figure 2c); Kerinci Seblat (1.07%, IUCN category II, Figure 2d); and Gumai Pasemah (0.27%, IUCN category IV, Figure 2e). In Kalimantan (Table S2), three hotspots (oscillating, sporadic, and consecutive) were identified inside two PAs: Kutai (oscillating–1.36%, IUCN category II, Figure 2f) and Teluk Kelumpang Selat Laut Selat Sebuku (consecutive – 8.51%; oscillating – 3.90%; sporadic – 2.49%, IUCN category Ia, Fig, 2g). More consecutive hotspots were found in the strict nature reserve Teluk Kelumpang Selat Laut Selat Sebuku (Figure 2g), suggesting an increase of forest loss was occurring at these locations in the recent years, despite the strict control of anthropogenic activities within the nature reserve.

### 3.3 Interplay between anthropogenic drivers of forest loss and deforestation hotspots

**FIGURE 3:**
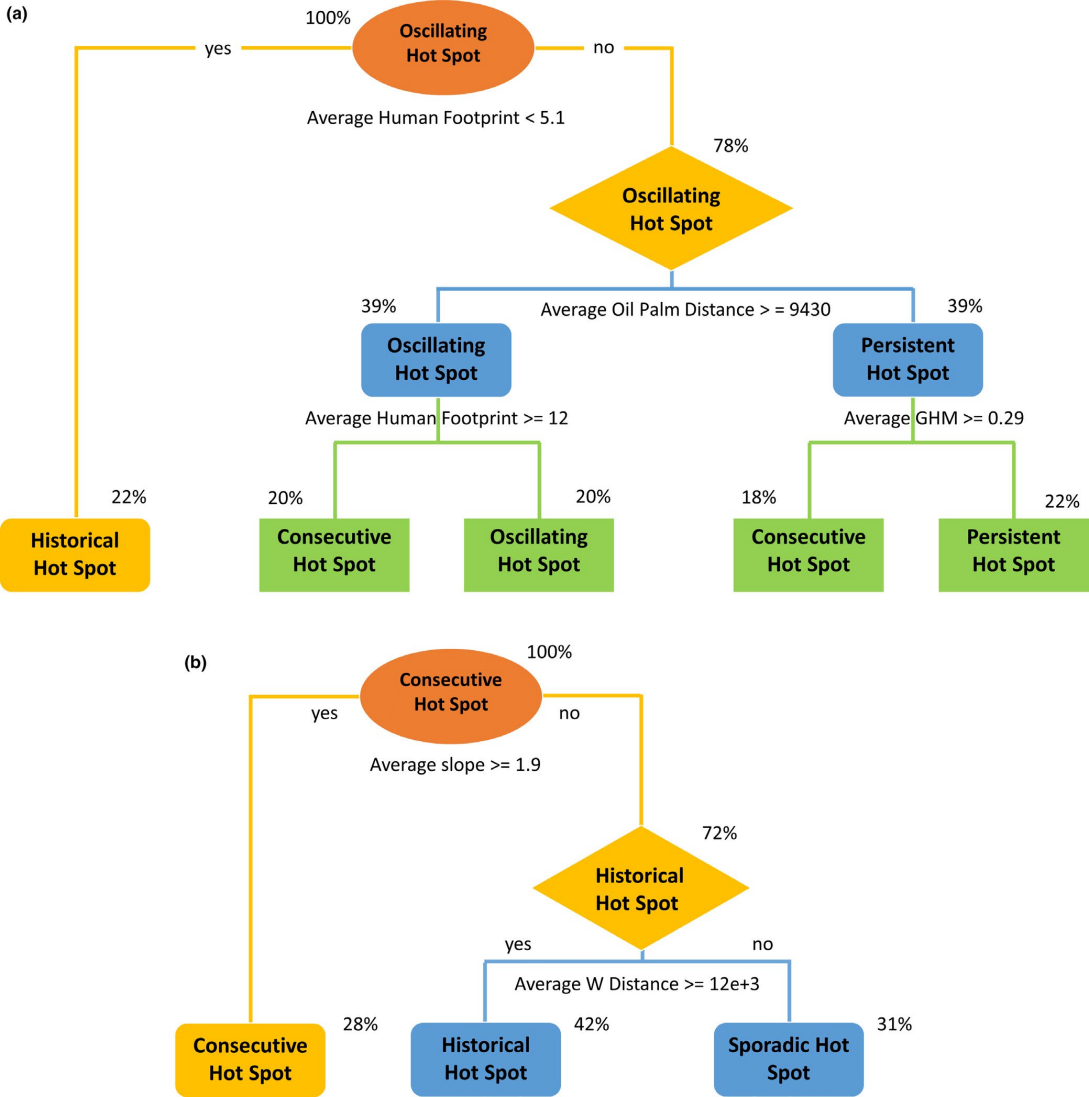
Decision trees in (a) Sumatra and (b) Kalimantan

Decision trees identified the most important drivers of deforestation in Sumatra: average human footprint, average wood fiber, and average oil palm distance (see Supporting Information). How these predictors contribute to the formation of different hotspots has been displayed in Figure 3a. For Kalimantan, the most important variables were average slope and average wood fiber plantation distance (see Supporting Information). Their contribution toward the formation of different hotspots has been displayed in Figure 3b. The percentages shown in the figures indicate how much of the evaluated data fall on each category.

The average human footprint values of less than 5.1 corresponded with historical deforestation hotspots. Average human footprint values of greater than 5.1 and oil palm plantation distance greater than 9,430 m corresponded with persistent deforestation hotspots. Average human footprint values greater than or equal to 12 resulted in consecutive deforestation hotspots. Consecutive deforestation hotspots had a higher average human footprint (13.3 ± 1.34) than intensifying and oscillating hotspots (8.75 ± 1.53 and 10.6 ± 0.70, respectively) (Table 3). Consecutive and intensifying deforestation hotspots were located in areas with an average elevation of approximately 100 m while sporadic hotspots were located in areas with an average elevation of 86‐m (Table 3).

**TABLE 3:** Human footprints and topographic characteristics in the deforestation hotspots in Sumatra (mean ± standard error of the mean)

Category	Human footprint	Elevation [m]	Slope [degrees]	Distance from oil palm plantation [m]	Distance from wood fiber plantation [m]
New hotspot	13.2 ± 1.04	105.0 ± 37.4	2.03 ± 0.52	23,984 ± 6,251	21,364 ± 5,500
Consecutive hotspot	13.3 ± 1.34	102.0 ± 30.4	2.74 ± 0.89	22,586 ± 7,444	35,287 ± 10,585
Intensifying hotspot	8.75 ± 1.53	100.0 ± 15.0	4.40 ± 0.88	2,800 ± 594	53,982 ± 722
Persistent hotspot	10.0 ± 1.17	51.6 ± 11.0	2.35 ± 0.47	3,184 ± 1,174	30,296 ± 8,416
Diminishing hotspot	3.83 ± 0.68	26.7 ± 3.2	0.82 ± 0.21	64 ± 52	2,663 ± 1545
Sporadic hotspot	11.5 ± 1.31	187.0 ± 118.9	2.28 ± 0.68	14,730 ± 7,302	26,222 ± 9,601
Oscillating hotspot	10.6 ± 0.70	86.2 ± 48.0	2.25 ± 0.93	18,362 ± 4,655	22,256 ± 5,819
Historical hotspot	2.88 ± 0.57	20.4 ± 1.89	0.82 ± 0.05	8,190 ± 2,022	None

In Kalimantan, average slope values greater than 1.9 corresponded with consecutive deforestation hotspots. Average slope values less than 1.9 corresponded with historical hotspots. Additionally, slope values less than 1.9 and higher than 12,000 m for average wood fiber plantation distance corresponded to sporadic deforestation hotspots (Figure 3b). The average human footprint and elevation values across all the hotspot categories were lower than Sumatra (Table 4).

**TABLE 4:** Human footprints and topographic characteristics in the deforestation hotspots in Kalimantan (mean ± standard error of the mean)

Category	Human footprint	Elevation [m]	Slope [degrees]	Distance from oil palm plantation [m]	Distance from wood fiber plantation [m]
Consecutive hotspot	6.59 ± 1.22	54.9 ± 9.7	2.12 ± 0.39	7,507 ± 1935	30,486 ± 15,134
Sporadic hotspot	7.93 ± 1.04	52.2 ± 9.7	1.11 ± 0.15	3,751 ± 1904	16,171 ± 10,600
Oscillating hotspot	6.43 ± 1.24	67.9 ± 11.6	1.95 ± 0.49	4,726 ± 1578	37,834 ± 14,962
Historical hotspot	6.78 ± 0.78	41.8 ± 3.7	1.53 ± 0.10	1709 ± 438	16,122 ± 2,137

The Kruskal–Wallis test on the five variables (human footprint, elevation, slope, and distances from oil palm and wood fiber plantations) showed no significant differences (with respective *p*‐values = .6842, .185, .1375, .0713, .3587) between groups in the data in Kalimantan, while in Sumatra, distances from Wood Fiber and Oil Palm Plantations as well as Human Footprint are significantly different at 5% significant level.

## 4 DISCUSSION

We examined how local anthropogenic and topographic characteristics affect the deforestation in Sumatra and Kalimantan using emerging hotspot analysis.

### 4.1 Effects of anthropogenic pressures

**FIGURE 4:**
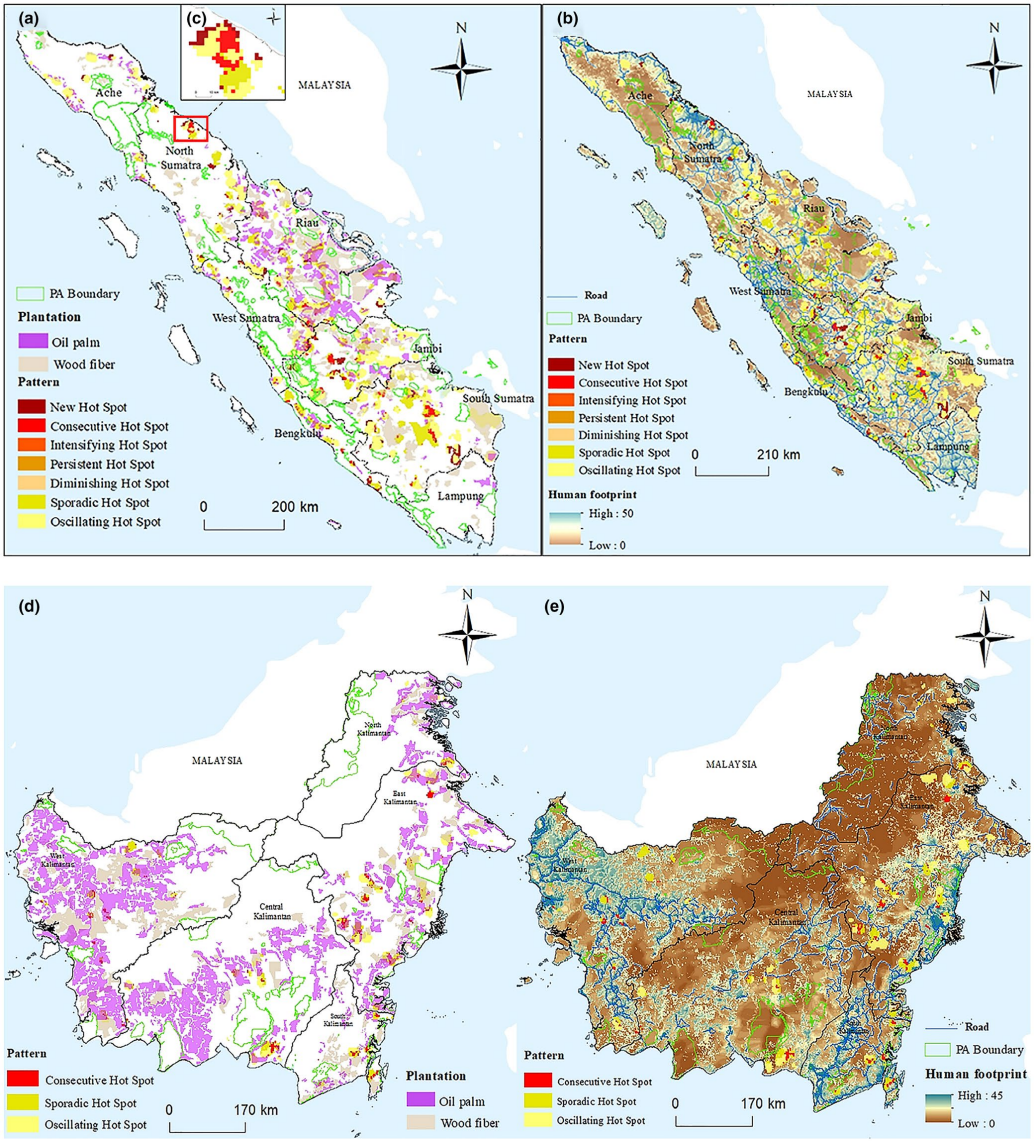
Distribution of oil palm and wood fiber plantations across (a) Sumatra and (d) Kalimantan. (b) Human pressure on the environment in (b) Sumatra and (e) Kalimantan

Within Sumatra, the majority of the deforestation hotspots were detected in central and southern areas, covering three provinces: Riau, Jambi, and South Sumatra. Most hotspots occurred in areas with higher human footprint. In Bengkulu, Jambi, and South Sumatra, new hotspots were observed surrounding roads (far from oil palm or wood fiber plantations), where accumulative human pressure on the environment was higher. Similarly, the hotspots found were mainly concentrated in the eastern and southern coastal areas of Kalimantan (Figure 4b). It may be argued that higher human effect, which manifests itself in the form increased road construction, croplands, and population density (Venter et al., 2016a, 2016b), has played a significant role in the spatial distribution of hotspots. Fiber plantation and logging concessions are responsible for the largest forest loss (~1.9 and ~1.8Mha, respectively) in Kalimantan, Sumatra, Papua, Sulawesi, and the Moluccas during 2000–2010, followed by oil palm plantations (Basyuni et al., 2018). However, land cover change in parts of Indonesia (e.g., Kalimantan) is a dynamic and multi‐trajectory phenomenon, involving the conversion of forests to croplands and smaller agricultural holdings (including rubber plantations) and subsequent conversion to large‐scale cash crop plantations such as fiber and oil palm (Van der Laan et al., 2018).

In the recent past, Eastern Kalimantan's high rates of deforestation have been drawn by large‐scale resource extraction, including the establishment of agricultural plantations (Dennis & Colfer, 2006; Dewi et al., 2005) and elevational profiles (Harris et al., 2008). Forests at lower elevations, including those in protected areas, are vulnerable to conversion to oil palm plantations and other agricultural uses (Fawzi et al., 2018). Eastern Kalimantan had among the highest levels of deforestation on the island in the late 1990s (Fuller et al., 2004). Eastern Kalimantan experienced high levels of transmigration during the 1970s and 1980s (Brookfield & Byron, 1990) which arguably has contributed to higher human pressures in the region.

In addition to Eastern Kalimantan, the plantations were found mainly in Western Kalimantan and the southwestern part of Central Kalimantan, while others along the eastern coastal areas. Like eastern Kalimantan, western Kalimantan too experienced high levels of transmigration which arguably contributed to higher anthropogenic pressures (Brookfield & Byron, 1990). Over the past few years, plantation expansion has leveled off in Sumatra, resulting in a decline in oil palm driven deforestation, while oil palm production shifted to Kalimantan, where it drove increasing deforestation (Austin et al., 2017). Sumatra's deforestation rates vary across its different administrative units. While Lampung had only a 3.74% rate of forest loss from 2000–12, Riau lost nearly 50% of its forest cover in the same time. High rates of deforestation in different parts are driven by logging and conversion to plantations (Supriatna et al., 2017).

Only a few hotspots have been observed within PAs in both Sumatra and Kalimantan, and these few all appeared at PA boundaries (Figure 2). This result is expected because it has been previously reported that the establishment of tropical PAs can lead to more forest loss in adjacent PAs, known as “neighborhood leakage” (Gaveau et al., 2009). One possible reason for this effect is that anthropogenic pressures develop along the edges of PAs, which spill into PAs (Figure 4b and 4e) (Harris et al., 2008). These in turn can arguably imperil PAs. While deforestation rates are relatively lower within Sumatra's PAs, wood plantations are still an important driver of forest loss. Expansion of timber plantations in and around PAs once the existing concessions are exhausted has been projected to drive up deforestation in Sumatra's PAs (Brun et al., 2015). For instance, the conversion to oil palm plantations has been identified as a driver of forest loss both outside and within the boundaries of the Gunung Leuser National Park in Sumatra (Supriatna et al., 2017). Similarly, deforestation has occurred both outside and within the borders of Kalimantan's PAs (Krasovskii et al., 2018). These too are threatened by illegal logging and oil palm expansion (Nellemann, 2007).

### 4.2 Effect of topography

Topographic characteristics such as elevation and slope were shown to protect the forest from loss. In both Sumatra and Kalimantan, hotspots were mainly distributed in areas of lower elevations or slopes, for example, the central region of Sumatra. This result is consistent with previous research showing that areas with relatively higher elevation and slope tend to remain forested (Brun et al., 2015; Poor, Frimpong, et al., 2019; Poor, Jati, et al., 2019). Human footprint values were lower in regions with high elevation or slope (Figures 3b and 4b), which may suggest that human activities are restricted there. Forest areas with these features can increase transport costs, requiring longer road lengths and more fuel consumption, thus reducing the efficiency of access to forest resources (Brun et al., 2015). Additionally, conversion from forests to agricultural lands in high‐elevation areas is usually less desirable, since higher altitude can lead to lower agricultural yields (Joppa & Pfaff, 2009).

### 4.3 Implications for conservation management

Emerging hotspot analysis was used in this research to evaluate statistically significant high clustering of forest loss, resulting in different categories of the hotspot (Table 2). Based on the definition of each category, locations showing intensifying and persistent hotspots are suggested for consideration as priority conservation areas—these areas have been detected as hotspots for forest loss for more than 16 years of the study period. The intensity of forest loss in intensifying hotspots showed an increasing trend during the study period, which suggests that intensifying hotspots should also be considered high‐priority locations for conservation. Establishing new PAs in these deforestation hotspots could bring benefits to local biodiversity by restricting human activities such as hunting. However, the implementation may be difficult, because there will be a demand for land as compensation for establishing Pas, and the cost can be high (Sanchez‐Cuervo & Aide, 2013).

In addition to intensifying and persistent hotspots, locations with new or consecutive hotspots should also be considered as targets for conservation. Hotspots in both of these categories appeared at these locations in 2018 and are showing an increasing trend (Table 2). Intensifying hotspots have been observed in certain PAs (e.g., Tesso Nilo National Park). Locations with new or consecutive hotspots were detected outside or at PA boundaries and were found in multiple provinces in both Sumatra and Kalimantan. There is a possibility that these locations may continue to experience higher deforestation than surrounding areas in the future. Emerging hotspot analysis can be performed annually to determine whether these locations remain hotspots. If so, effective management actions relating to population control will be required to reduce human pressure near PA boundaries (Joppa & Pfaff, 2009).

### 4.4 Comparison of emerging hotspots

On the basis of GFC data for 2000–2018, extensive areas of new hotspots were identified in central Sumatra and western and eastern Kalimantan. Previous research (conducted using GFC data from 2000–2014) discovered persistent hotspots in Riau, Sumatra, and Central Kalimantan, Kalimantan (Harris et al., 2017). In the present study, new hotspots were detected only in Sumatra, and these covered small areas. Persistent and intensifying hotspots made up only 0.09% of the total hotspots detected in Sumatra, fewer than those detected in previous research (Harris et al., 2017), and none were observed in Kalimantan. Furthermore, this research identified vast areas in Sumatra with sporadic and oscillating hotspots, rather than new hotspots, and extensive areas were observed with no hotspots in Kalimantan.

It is possible that areas of forest loss have been reduced during 2014–2018, as the dominating hotspot has been converted from “new” for the study period 2001–2014 (Harris et al., 2017) to oscillating in 2001–2018. The reduction in new oil palm plantations on forested land may be the cause of the reduction in deforestation. Recent work has shown that the proportion of new plantations leading to forest loss declined from 22% from 2000–2010 to 18% during 2010–2015 (Austin et al., 2017).

### 4.5 Limitations and recommendations

It seems that the road network data used in this research were incomplete. Very few roads were shown within PAs, while previous research has shown an increasing trend of road length and density within PAs (Poor, Frimpong, et al., 2019; Poor, Jati, et al., 2019). It is possible that only major roads have been included in the dataset used, while other grades of roads, such as secondary roads or footpaths, are excluded (Poor, Frimpong, et al., 2019; Poor, Jati, et al., 2019). Thus, the road density within PAs might be underestimated in this work. Furthermore, the human footprint map used in this research shows accumulative human pressure on the environment as of 2009, which may not reflect current human footprint values. However, the 2009 human footprint map is the latest version available from SEDAC. Additionally, land‐use change data were not used in this research. Hotspots of deforestation have been observed to influence land‐use change, as increasing hotspots can cause an increase in agricultural land and a decline in woody or mixed woody vegetations (Sanchez‐Cuervo & Aide, 2013). In future research, locations with cold spots of land‐use change, such as abandoned croplands, can be used for reforestation by establishing PAs. Although initial biodiversity levels might be low at these lands, a long‐term benefit of conserving ecosystem services could result in biodiversity gains (Sanchez‐Cuervo & Aide, 2013).

Since the initial publication, the GFC data have been updated to improve the accuracy of detecting forest loss. The reprocessing of data started in 2011 and has not been implemented for years preceding 2011 (Hansen et al., 2013). The updated detection method is more sensitive to forest loss and, in particular, improves the detection of forest loss in areas where selective logging and short‐cycle plantation clearing are occurring. Consequently, additional forest loss has been detected for 2011–2018 in comparison to the original measurement for 2000–2010. A future version of GFC data (version 2.0) will update forest loss data preceding 2011 and keep the detection method consistent over the entire period 2000–2018. Therefore, it is recommended that emerging hotspot analysis should be performed on version 2.0 of the GFC data when it becomes available.

## 5 CONCLUSIONS

From the EHA performed to evaluate the spatial–temporal trends of forest loss in Sumatra and Kalimantan during the period 2001–2018, it was found that deforestation hotspots were mainly distributed outside PAs and occasionally on the boundaries but never in the core zones.

In Sumatra, seven deforestation hotspots (New, Consecutive, Intensifying, Persistent, Diminishing, Sporadic, and Oscillating) were detected in central and southern parts of Sumatra, mainly concentrated in the provinces of Riau, Jambi, and South Sumatra. Only three deforestation hotspots (Consecutive, Sporadic, and Oscillating) were detected in Kalimantan. Hotspots in Kalimantan were mainly observed in eastern and southern coastal areas, with some in western areas. The categories of hotspots observed also differed between the two islands. Four hotspot categories were detected only in Sumatra: New, Intensifying, Persistent, and Diminishing. New hotspots were observed in several provinces ranging from the north (e.g., Ache) to south (e.g., South Sumatra). Although different hotspot categories were detected on the two islands, oscillating hotspots dominated on both islands.

The distribution pattern of hotspots was influenced by both topographic and anthropogenic factors. The majority of hotspots are concentrated in areas with low elevation and high human pressure. Hotspots were only detected at PA boundaries, as these boundaries are usually located at areas of low elevation or slope. Higher human pressure was mainly observed along roads, locations where more hotspots could also be observed.

The results of this work emphasize specific areas of forest loss that should be considered as a conservation priority. Deforestation hotspots should be considered as priority conservation targets because these locations contain abundant biodiversity and are under high pressure for land conversion. These locations may initially have low‐level biodiversity but are expected to improve ecosystem services in the short to medium term and assist biodiversity recovery in the long term.

Future research should reapply EHA to data from this period when all GFC data have been reprocessed by the improved detection method. It is suggested that complementary approaches incorporating present land change dynamics should be included in the design of future PAs. Emerging hotspot analysis can also be applied to annual land‐use change data to generate cold spots of land‐use change where PAs can be implemented for reforestation.

## CONFLICTS OF INTERESTS

The authors declare that they have no competing interests.

## AUTHOR CONTRIBUTIONS

Siheng Yan: Data gathering and analysis. Minerva Singh: Advising and contributing extensively to the final written work, including undertaking significant rewrites as recommended by the reviewers. Minerva Singh: Machine learning and statistical analysis. All authors commented on the manuscript.

## Supporting information

Supplementary Material

## Data Availability

All data used in this study are from published studies or open access databases. For more details, please check https://doi.org/10.5061/dryad.kkwh70s49.
