# Supplementary material for: Spatial–temporal variations in deforestation hotspots in Sumatra and Kalimantan from 2001–2018
Source: Ecol Evol. 2021 May 2;11(12):7302–14. doi: 10.1002/ece3.7562 (PMC8216897; doi:10.1002/ece3.7562)
Supplement: Supplementary file 1 — Supplementary Material [file ECE3-11-7302-s001.docx]

**SUPPLEMENTARY MATERIALS**

**Table S1. Hot spot coverages in the Protected Areas in Sumatra.**

| **Gumai Pasemah, Sumatra** | | |
| --- | --- | --- |
| PATTERN | Area, km^2^ | Percent coverage |
| New Hot Spot | 19.2978 | 4.023137 |
| No Pattern Detected | 254.1319 | 52.98052 |
| **Oscillating Hot Spot** | **2.58515** | **0.538943** |
| Total Area | 479.6704 |  |

| **Gunung Leuser National Park, Sumatra** | | |
| --- | --- | --- |
| PATTERN | Area, km^2^ | Percent coverage |
| **New Hot Spot** | **22.63553** | **0.268334** |
| No Pattern Detected | 1702.837 | 20.18635 |

| **Kerinci Seblat, Sumatra** | | |
| --- | --- | --- |
| PATTERN | Area, km^2^ | Percent coverage |
| Consecutive Hot Spot | 39.17002 | 0.288032 |
| **New Hot Spot** | **145.3728** | **1.068979** |
| No Pattern Detected | 4490.361 | 33.01927 |
| **Oscillating Hot Spot** | **805.9468** | **5.926422** |
| **Sporadic Hot Spot** | **35.70818** | **0.262575** |
|  |  |  |

**S2. The Most Important Variables Driving the Spatial-Temporal Distribution of Deforestation Hotspots
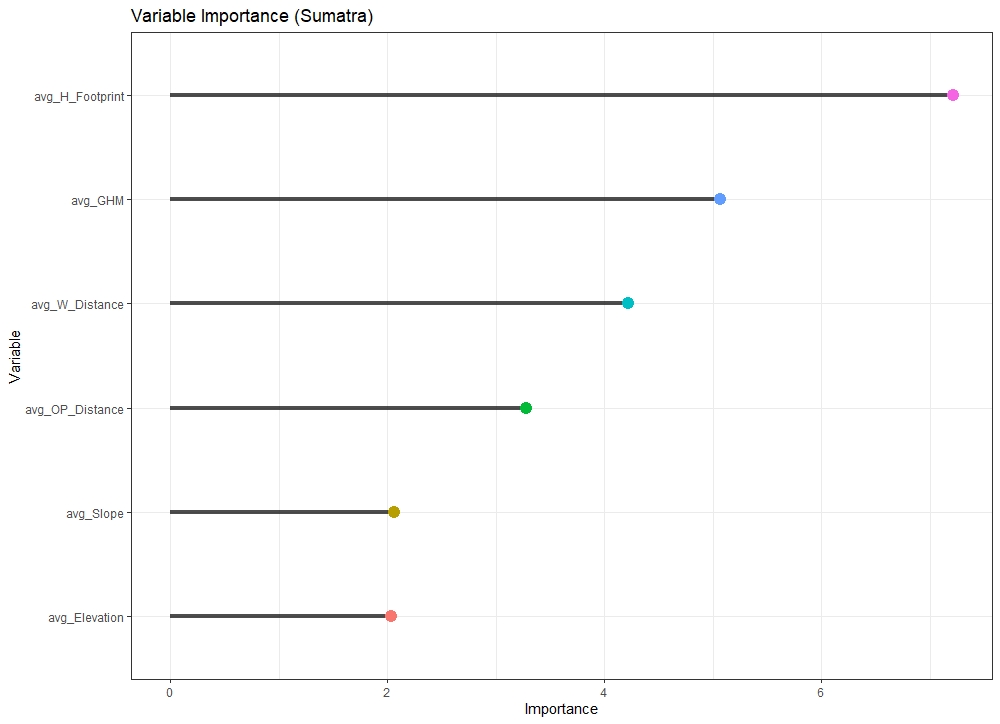
**

**
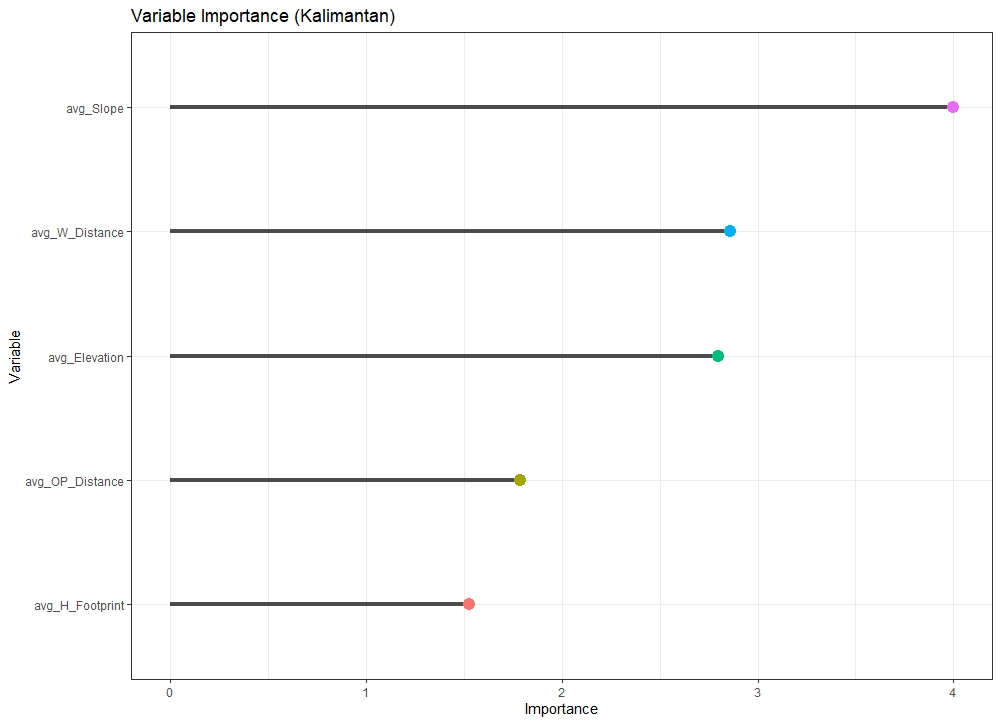
**

**Table S2. Hot spot coverages in the Protected Areas in Kalimantan.**

| **Teluk Kelumpang, Selat Laut dan Selat Sebuku, Kalimantan** | | |
| --- | --- | --- |
| PATTERN | Area, km^2^ | Percent coverage |
| **Consecutive Hot Spot** | **52.0382** | **8.510284** |
| No Pattern Detected | 471.2691 | 77.07095 |
| **Oscillating Hot Spot** | **23.85603** | **3.901395** |
| **Sporadic Hot Spot** | **15.22027** | **2.489111** |
|  | 611.4743 |  |

| **Kutai, Kalimantan** | | |
| --- | --- | --- |
| PATTERN | Area, km^2^ | Percent coverage |
| No Pattern Detected | 851.4804 | 42.22254 |
| **Oscillating Hot Spot** | **27.38521** | **1.357956** |

s
